# Health Impacts of Climate Change in Pacific Island Countries: A Regional Assessment of Vulnerabilities and Adaptation Priorities

**DOI:** 10.1289/ehp.1509756

**Published:** 2015-12-08

**Authors:** Lachlan McIver, Rokho Kim, Alistair Woodward, Simon Hales, Jeffery Spickett, Dianne Katscherian, Masahiro Hashizume, Yasushi Honda, Ho Kim, Steven Iddings, Jyotishma Naicker, Hilary Bambrick, Anthony J. McMichael, Kristie L. Ebi

**Affiliations:** 1 National Centre for Epidemiology and Population Health, Australian National University, Canberra, Australia; 2 Division of Pacific Technical Support, Western Pacific Regional Office, World Health Organization, Suva, Fiji; 3 School of Public Health, University of Auckland, Auckland, New Zealand; 4 Department of Public Health, University of Otago, Wellington, New Zealand; 5 World Health Organization Collaborating Centre for Environmental Health Impact Assessment, Curtin University, Perth, Australia; 6 Institute of Tropical Medicine, Nagasaki University, Nagasaki, Japan; 7 Faculty of Health and Sport Science, University of Tsukuba, Tsukuba, Japan; 8 Graduate School of Public Health, Seoul National University, Seoul, Republic of Korea; 9 Centre for Health Research, School of Medicine, University of Western Sydney, Sydney, Australia; 10 School of Public Health, University of Washington, Seattle, Washington, USA

## Abstract

**Background:**

Between 2010 and 2012, the World Health Organization Division of Pacific Technical Support led a regional climate change and health vulnerability assessment and adaptation planning project, in collaboration with health sector partners, in 13 Pacific island countries—Cook Islands, Federated States of Micronesia, Fiji, Kiribati, Marshall Islands, Nauru, Niue, Palau, Samoa, Solomon Islands, Tonga, Tuvalu, and Vanuatu.

**Objective:**

We assessed the vulnerabilities of Pacific island countries to the health impacts of climate change and planned adaptation strategies to minimize such threats to health.

**Methods:**

This assessment involved a combination of quantitative and qualitative techniques. The former included descriptive epidemiology, time series analyses, Poisson regression, and spatial modeling of climate and climate-sensitive disease data, in the few instances where this was possible; the latter included wide stakeholder consultations, iterative consensus building, and expert opinion. Vulnerabilities were ranked using a “likelihood versus impact” matrix, and adaptation strategies were prioritized and planned accordingly.

**Results:**

The highest-priority climate-sensitive health risks in Pacific island countries included trauma from extreme weather events, heat-related illnesses, compromised safety and security of water and food, vector-borne diseases, zoonoses, respiratory illnesses, psychosocial ill-health, non-communicable diseases, population pressures, and health system deficiencies. Adaptation strategies relating to these climate change and health risks could be clustered according to categories common to many countries in the Pacific region.

**Conclusion:**

Pacific island countries are among the most vulnerable in the world to the health impacts of climate change. This vulnerability is a function of their unique geographic, demographic, and socioeconomic characteristics combined with their exposure to changing weather patterns associated with climate change, the health risks entailed, and the limited capacity of the countries to manage and adapt in the face of such risks.

**Citation:**

McIver L, Kim R, Woodward A, Hales S, Spickett J, Katscherian D, Hashizume M, Honda Y, Kim H, Iddings S, Naicker J, Bambrick H, McMichael AJ, Ebi KL. 2016. Health impacts of climate change in Pacific island countries: a regional assessment of vulnerabilities and adaptation priorities. Environ Health Perspect 124:1707–1714; http://dx.doi.org/10.1289/ehp.1509756

## Introduction

Climate change is widely acknowledged to be one of the most serious global threats to future human population health and international development (Costello et al. 2009; Stephenson et al. 2013; Woodward et al. 2014). The Fifth Assessment Report (AR5) from the Intergovernmental Panel on Climate Change (IPCC) affirms that recent decades have seen warming air and ocean temperatures, altered precipitation patterns, rising sea levels, and changes in the frequency and intensity of some extreme events such as droughts, floods, and storms (IPCC 2014). The AR5 also asserts with greater confidence than reported in 2007 (Parry et al. 2007) that recent warming is largely attributable to human activity. Further, there is increasing certainty that these trends will continue or, in some cases, accelerate (IPCC 2014).

A changing climate has significant and diverse impacts on human health (McMichael and Lindgren 2011; Woodward et al. 2014). The pathways by which climate change affects health vary according to their modes of action and include primary or direct effects (e.g., injuries and deaths caused by extreme weather events such as cyclones), secondary or indirect effects (e.g., the increasing geographic range of, and population exposed to, vectors that spread disease), and tertiary, diffuse, and/or delayed effects (e.g., disruptions to health and social services) (Butler and Harley 2010; McMichael 2013).

**Figure 1:**
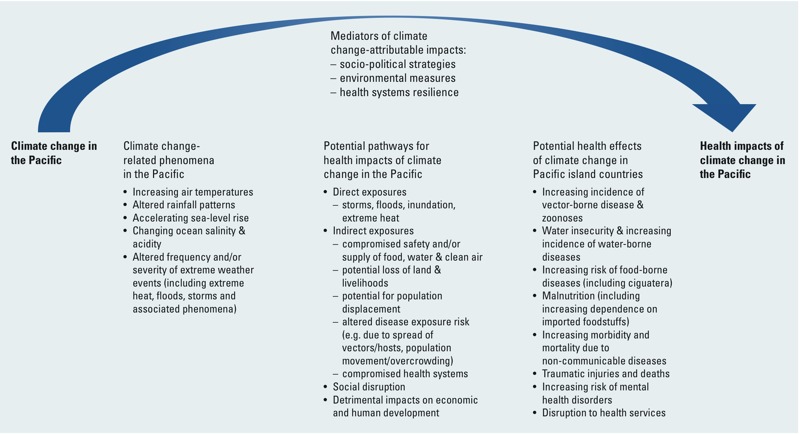
Climate change and health impact pathways relevant to Pacific island countries.

Pacific island countries (PICs) are among those most vulnerable to the health impacts of a changing climate (Hanna and McIver 2014; Woodward et al. 2000). This vulnerability is a function of their exposure to changing weather patterns associated with climate change, the health risks entailed, and the limited capacity of these countries to manage and adapt in the face of such risks. The climate change phenomena occurring in the Pacific pose a suite of health hazards to the island communities across the region. A conceptualization of the pathways by which climate change will affect health in the Pacific and the major anticipated impacts throughout the region is shown in Figure 1.

In PICs, this vulnerability reflects the unique geographic, demographic, and socioeconomic characteristics of small island developing states (SIDS) (Table 1), which, combined with their contemporary burden of ill-health and relatively low health systems capacity, give credence to the epithet “canaries in the coalmine” of climate change and health (Hanna and McIver 2014).

**Table 1:** Characteristics of Pacific island countries.

Pacific island country	Geography		Demography		Economy	
	Land area (km^2^)^*a*^	Max elevation (m)^*b*^	Population^*b*^	Population density (per km^2^)	Per capita GDP^*b*^^,^^*c*^	Main industries # (% GDP)^*b*^
Cook Islands	240	652	15,000	42	9,100	T
Federated States of Micronesia	700	791	112,000	158	2,200	A, F
Fiji	18,000	1,324	868,000	47	4,600	T, A
Kiribati	800	3, 81^*d*^	101,000	135	6,200	A, F, R
Marshall Islands	200	10	64,000	342	2,500	Aid (US)
Nauru	20	71	10,000	480	5,000	M
Niue	260	68	1,000	5	8,400	Aid (NZ)
Palau	500	242	21,000	39	10,000	T, A, F
Samoa	2,900	1,857	184,000	63	6,000	R, T, A, F
Solomon Islands	28,000	2,335	552,000	18	3,300	A, F, forestry
Tonga	750	1,033	105,000	139	7,500	A, F
Tuvalu	300	5	10,000	476	3,400	R, trusts, A
Vanuatu	12,000	1,877	246,000	20	2,700	A, F, T
Abbreviations: A, agriculture; F, fishing; M, mining; NZ, New Zealand; R, remittances; T, tourism; US, United States. All data are for 2011 unless otherwise stated. ^***a***^UN Office of the High Representative for the Least Developed Countries, Landlocked Countries and Small Island Developing States (UN-OHRLLS 2015). ^***b***^Central Intelligence Agency (2012). ^***c***^Per capita gross domestic product based on purchasing power parity. Estimates are for 2011 except for Marshall Islands (year 2008), Nauru (year 2005), Palau (year 2009), Tuvalu (year 2010), and Vanuatu (year 2009).^***d***^Elevations for South Tarawa (the capital atoll of Kiribati) and Banaba (an outlying atoll), respectively.						

Recognizing the risks to health posed by climate change, the World Health Organization (WHO) Regional Offices for the Western Pacific and South-East Asia issued a joint *Regional Framework for Action to Protect Human Health from the Effects of Climate Change in the Asia-Pacific Region* (WHO 2007; Hanna and McIver 2014) This framework committed all countries in the region to increasing awareness of climate change and health, to strengthening the capacity of health systems to protect against climate-related health risks and reduce greenhouse gas emissions in the health sector, and to ensuring that health concerns were addressed in climate action in other sectors. Specific actions mandated in the framework included supporting formalized climate change and health vulnerability assessments and leading the health sector’s contribution to national adaptation planning processes in the region.

Subsequently, the health ministers of PICs strengthened their commitments to action on climate change at their biennial meeting in Madang, Papua New Guinea, in 2009. The *Madang Commitment* included further recommendations related to vulnerability assessments and adaptation planning, framing these within the *Healthy Islands* vision for health systems development in the Pacific (Galea et al. 2000; WHO 2009).

**Figure 2:**
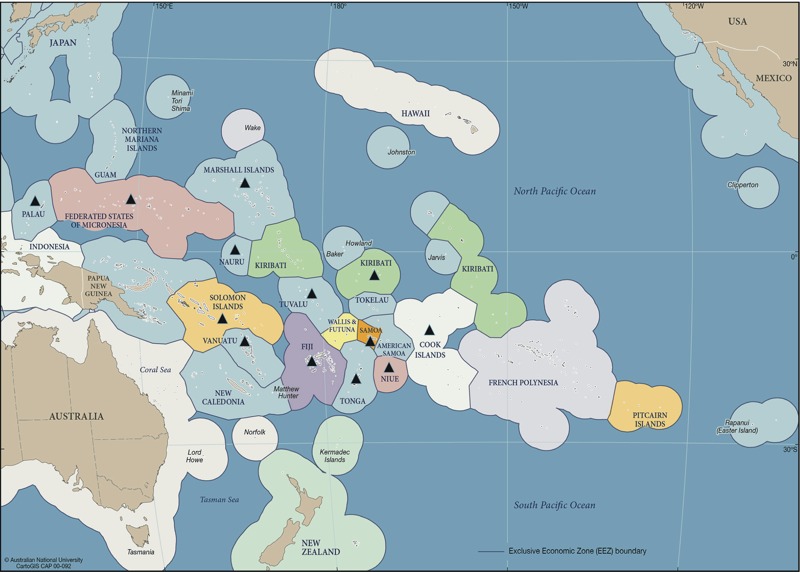
Countries involved in WHO-supported climate change and health project in the Pacific (2010–2013) [Source: adapted from CartoGIS (2016), College of Asia and Pacific, The Australian National University, under terms of ShareAlike 3.0 Australia (https://creativecommons.org/licenses/by-sa/3.0/au/legalcode). Some changes have been made to this figure: black triangles were added to indicate the project countries].

This paper describes the process and outcomes of climate change and health vulnerability assessments in 13 SIDS in the Pacific region: Cook Islands, Federated States of Micronesia, Fiji, Kiribati, Marshall Islands, Nauru, Niue, Palau, Samoa, Solomon Islands, Tonga, Tuvalu, and Vanuatu (see map in Figure 2).

In this paper, we report how these assessments link with adaptation planning; we also highlight some of the unique challenges facing PICs in the context of climate change, and we summarize the corresponding recommendations arising from the regional project. This paper is aimed at a general scientific audience and is a synthesis of the key technical findings and policy implications of the forthcoming WHO report entitled, *Human Health and Climate Change in Pacific Island Countries* (McIver et al. 2015b).

## Methods

Between 2010 and 2012, the WHO Division of Pacific Technical Support, with support from the Western Pacific Regional Office (WPRO) and funding from the governments of the Republic of Korea and Japan, led a regional climate change and health vulnerability and adaptation project involving 11 PICs: Cook Islands, Federated States of Micronesia (FSM), Kiribati, Marshall Islands, Nauru, Niue, Palau, Solomon Islands, Tonga, Tuvalu and Vanuatu. This project was implemented in three phases, with the 11 countries divided into three groups along broadly geographic lines and a team of expert climate change and health consultants guiding and assisting each group. These 11 countries form the majority of independent or autonomous states in the Pacific region, along with Fiji and Samoa, which carried out related projects (see below). Papua New Guinea has been involved in a separate climate change and health project, along with other countries in the Western Pacific region, and was not included; the French territories of New Caledonia, French Polynesia, and Wallis and Futuna were excluded for similar reasons.

In the first phase, sub-regional inception meetings were held in Auckland, New Zealand (for the group that included Cook Islands, Kiribati, Niue, Tonga, and Tuvalu); Honiara, Solomon Islands (for Nauru, Solomon Islands, and Vanuatu); and Pohnpei, FSM (for FSM, Marshall Islands, and Palau). During these meetings, the science of climate change and health was reviewed, along with the relevant work previously conducted in each country, and plans were made for the in-country vulnerability assessment and adaptation planning phases of the project.

The second phase involved a mixed-methods approach to ascertain each country’s climate-sensitive health risks based on a combination of review and analysis of climate and health data, stakeholder consultations, and an assessment of the potential impacts of a changing climate across different aspects of society. Where possible, epidemiological analysis was performed on the available data for historical climate variables and climate-sensitive diseases (e.g., diarrheal disease, dengue fever, and leptospirosis). Performing these analyses was possible only in countries with adequate data (in terms of quality and quantity) and available technical support. The sophistication of the modeling undertaken ranged from simple reviews of disease burdens and weather patterns in Kiribati (McIver et al. 2014) to Poisson regression models in FSM (McIver et al. 2015a) and similar techniques, combined with spatial modeling, for multiple climate-sensitive diseases in Fiji (McIver et al. 2012). The results of these country-specific analyses have not been included in this paper, which instead provides a more general, regional overview.

In the final phase, each country’s climate change and health vulnerabilities were assessed using a “likelihood-versus-consequence” matrix. This tool was used to rank climate-sensitive health priorities, rationalize resources, and focus the activities of the health sector on the most urgent adaptation activities. Its use was based on precedents in Australia (Brown et al. 2014) and on growing evidence of its utility in this context in Pacific island countries such as Vanuatu (Spickett and Katscherian 2014; Spickett et al. 2013). In using this matrix, each climate-sensitive health risk was considered in terms of *a*) the likelihood of the burden of disease increasing with climate change (over a 20-year time-frame), *b*) a range of climate change projections (as localized as possible), and *c*) the impact of such an increased health burden occurring (considering the resilience or coping capacity of the community and health sector to manage such consequences).

Each Pacific island country differed in terms of its willingness and perceived capacity to manage its respective highest-priority climate-sensitive health risks. Some elected to include most or all of these hazards in their adaptation plans; others chose to concentrate on the few health impacts deemed to represent the greatest threat according to the abovementioned matrix. Thus, the climate-sensitive health risks presented in the “Results” below should be considered a synthesis of each country’s priorities rather than a true cross-country comparison of risks.

Following the prioritization of these climate change–related health vulnerabilities, relevant adaptation strategies were planned accordingly. Adaptation measures were categorized as follows: legislative and regulatory, public education and communication, surveillance and monitoring, ecosystem intervention, infrastructure development, technological and engineering responses, medical intervention, and research.

Each adaptation measure was prioritized for each PIC according to its local relevance, the current capacity of the health system, the inclusion of vulnerable groups, the manner in which adaptations could be implemented, and the identification of sectors that would be involved in the development and implementation of the adaptation strategies. The country teams—which were typically, but not exclusively, led by the Ministry of Health or its equivalent in collaboration with other government departments, civil society organizations and major private sector stakeholders—chose the factors that would be included and decided upon their relative weighting.

These vulnerabilities and the responses required from the health sector were incorporated into National Climate Change and Health Action Plans (NCCHAPs) or an equivalent thereof. At present, these domestic policy documents are in various stages of finalization and implementation across the Pacific island countries participating in this regional project.

The vulnerability assessment and adaptation planning process was slightly different for Samoa. An initial workshop on health aspects of vulnerability and adaptation to climate change was conducted in Apia in 2010 as part of a national project entitled, “Integrating Climate Change Risks in the Agriculture and Health Sectors in Samoa.” Building on this work, a climate change and health adaptation strategy and action plan was developed for Samoa in 2013.

The approach was different again for Fiji, which since 2010 has been participating in a global climate change and health adaptation pilot project led by WHO with support from the United Nations Development Programme (UNDP) and with funding from the Global Environment Facility (GEF; https://www.thegef.org/). In Fiji’s *Piloting Climate Change Adaptation to Protect Human Health* project, a small number of high-priority climate-sensitive diseases were selected as the focus for the vulnerability assessment, research, capacity-building, community education, and adaptation elements of the project (McIver et al. 2012).

The process outlined above broadly followed the guidelines laid out by WHO for vulnerability assessment and adaptation planning (Kovats et al. 2003; WHO 2013b). Table 2 compares the WHO theoretical framework with the actual steps implemented in the abovementioned PICs.

**Table 2:** Steps involved in vulnerability assessment and adaptation planning process in Pacific island countries (PICs) and comparison with WHO framework [the latter adapated from Kovats et al. (2003)].

WHO framework for vulnerability assessment and adaptation planning	Vulnerability assessment and adaptation planning process implemented in PICs
Determine scope of assessment	Project designed and resourced Eleven PICs divided into three regions along roughly geographic and cultural lines Expert technical guidance provided to each group Inception meetings held and work plans made for each country
Describe current distribution and burden of climate-sensitive diseases	Available information and data on climate and climate-sensitive diseases reviewed and described in each country Environmental epidemiological analysis undertaken where possible
Identify and describe current strategies, policies and measures that reduce the burden of climate-sensitive diseases	Health sector and other relevant policies (e.g., climate change policies, strategic development plans) reviewed and linked with health adaptation planning
Review the health implications of the potential impact of climate variability and change on other sectors	Wide stakeholder, cross-sectoral engagement ensured in health adaptation planning
Estimate the future potential health impact using scenarios of future climate change, population growth and other factors and describe the uncertainty	Some modeling of future climate change-attributable burden of disease attempted; limited by lack of downscaled climate projections and sufficient quantity and quality of data on climate-sensitive diseases
Synthesize the results and draft a scientific assessment report	National Climate Change and Health Action Plans (NCCHAPs)—or equivalent—prepared for each of the 11 PICs
Identify additional adaptation policies and measures to reduce potential negative health effects, including procedures for evaluation after implementation	Adaptation strategies prioritized Highest priority adaptations commenced in some PICs (Table 3) Guidance provided to countries on methods for iterative information management, monitoring, and evaluation
Source: adapted from WHO (2013a). Protecting Health from Climate Change: Vulnerability and Adaptation Assessment. http://www.who.int/globalchange/publications/vulnerability-adaptation/en/.	

In each of the 13 PICs, efforts were made to include mechanisms for monitoring and evaluation and iterative information management—for example, the incorporation of updated data on climate and climate-sensitive diseases—into each NCCHAP (Ebi 2014).

## Results

Table 3 summarizes the climate-sensitive health risks prioritized in each country’s vulnerability assessment. These risks are subdivided into three categories: direct, indirect, and diffuse effects, in accordance with the international nomenclature (McMichael and Lindgren 2011). Examples of direct effects include the traumatic injuries and deaths that occur during hydro-meteorological disasters and the detrimental physiological consequences of heatwaves. Indirect effects occur through disruption of ecological systems; examples include increased pathogen loads in food and water in hotter and/or more humid conditions and altered geographic ranges and biting habits of mosquitoes that spread diseases such as malaria and dengue fever. Diffuse effects are related to societal dysfunction, key examples of which are disrupted health services, population displacement, and potential conflict over climate-related resources (Kjellstrom and McMichael 2013).

**Table 3:** Highest priority climate-sensitive health risks in individual Pacific island countries (with each country’s highest priorities indicated by “x”).

Climate-sensitive health risk	Country												
	Cook Islands	FSM	Fiji	Kiribati	Marshall Islands	Nauru	Niue	Palau	Samoa	Solomon Islands	Tonga	Tuvalu	Vanuatu
Direct effects
Health impacts of extreme weather events^*a*^	x	x			x	x	x	x	x	x	x	x	x
Heat-related illness^*b*^	x					x	x			x			x
Indirect effects
Water security & safety (including water-borne diseases)^*c*^	x	x	x	x	x	x	x	x	x	x	x	x	x
Food security & safety (including malnutrition & food-borne diseases)^*d*^	x	x	x	x	x	x	x		x	x	x	x	x
Vector-borne diseases^*e*^	x	x	x	x	x	x	x	x	x	x	x	x	x
Zoonoses^*f*^		x	x					x
Respiratory illness^*g*^	x	x			x	x	x	x		x		x	x
Disorders of the eyes, ears, skin, and other body systems^*h*^			x		x		x			x		x	x
Diffuse effects
Disorders of mental/psycho-social health^*i*^		x	x		x	x		x		x		x	x
Non-communicable diseases (NCDs)^*j*^		x	x		x		x	x	x	x	x	x	x
Health system deficiencies^*k*^			x	x
Population pressures^*l*^				x
FSM, Federated States of Micronesia. A number of climate-sensitive health risks may be considered to cut across categories: for example, there may be direct mental health consequences of extreme weather events; NCDs may be affected indirectly through disruption of food supplies, or more diffusely through socio-political strategies related to climate change, industry, and trade; health systems problems may be directly affected by extreme weather events as well as via the broader impact of climate change on development. ^***a***^This was typically taken to mean traumatic injuries and deaths, but may also be understood to include the psychosocial impacts of extreme events. ^***b***^Including occupational exposure to hotter working conditions. ^***c***^This category encompasses water-borne infections causing diarrheal illness, as well as typhoid fever, and also includes problems such as sea-level rise-induced salination of potable water supplies. ^***d***^Including food insecurity, food-borne diseases causing diarrheal illness, and ciguatera (“fish poisoning”). ^***e***^Including, but not limited to, dengue fever and malaria; noting that these two diseases occur in some, but not all, PICs (of those countries listed, malaria is currently limited to Solomon Islands and Vanuatu). ^***f***^The primary zoonosis of concern in most PICs is leptospirosis. ^***g***^Including infections, obstructive airways disease (e.g., asthma), and the pulmonary effects of heat and air pollution. ^***h***^This category includes a range of health problems, from skin infections and cataracts to sexually transmitted infections that were of concern in various PICs in the context of climate change. ^***i***^Includes the unspecified detrimental effects of social disruption (e.g., loss of life, land, or livelihoods) because of climate change–related phenomena; this category may include, *inter alia*, depression, anxiety, and posttraumatic stress disorder. ^***j***^In this context, NCDs refer primarily to circulatory diseases (e.g., cardiovascular disease, cerebrovascular diseases, hypertension) as well as to diabetes; in some PICs, this was also taken to include cancers and mental health disorders. ^***k***^Including compromised access to health services, damage to health infrastructure, and additional strains on scarce resources (e.g., for climate-sensitive disease surveillance). ^***l***^Includes the possibility of climate change–induced resettlement and the effects of climate change–induced sea-level rise in exacerbating overcrowding.													

This list was not intended to be comprehensive, to describe every conceivable risk to health that can be attributed to climate change; only those risks regarded by the country teams as being most important at the present time are included in this summary table. This table is also not intended to serve as a tool for comparison because although all countries used a similar process of prioritizing climate change and health vulnerabilities, each country differed in terms of the number of these hazards it felt appropriate to address in its respective adaptation plans. Thus, the absence of an entry in a row for a particular country in Table 3 should not necessarily be interpreted to mean that the country did not perceive that specific climate-sensitive health risk to be a problem; rather, this health risk was not among the most immediate priorities for that country at that time.

Table 3 displays some common themes in terms of climate-sensitive health risks across the Pacific. Climate change–attributable impacts on extreme weather events and diseases related to food, water, and vectors are prominent concerns throughout the region. Specific diseases such as dengue fever, malaria, diarrheal illness, leptospirosis, typhoid fever, respiratory infections, obstructive airways disease, and malnutrition are generally considered to be highly climate-sensitive (Woodward et al. 2014). There is thus a clear and relatively urgent need for these and other hazards (such as the health effects of heat and extreme weather events) to be considered in the context of climate change in the Pacific and to be anticipated accordingly (Haines et al. 2014).

However, there are some climate-related health risks that are of concern in the Pacific to an extent not documented elsewhere in the world: notably, noncommunicable diseases (NCDs), disorders of mental/psychosocial health, and ciguatera (Mannava et al. 2015; WHO 2013c). The potential for climate change to amplify the drivers of NCD risk in the Pacific is considered in more detail below.

In addition, there are other important aspects of health vulnerability in the region that are unique to, or at least uniquely highly prioritized in, a small number of PICs. These aspects include high fertility rates and overcrowding in atoll nations such as Kiribati which, combined with limited land area, low elevation, and the threat posed by rising seas, may lead to forced relocation, which brings with it a particular suite of health complications (McMichael et al. 2012; Berry et al. 2010).

Women and children are expected to experience a disproportionate burden of climate change and health impacts in the Pacific (Lawler 2011) and elsewhere, particularly in the developing world (IPCC 2014).

With respect to adaptation, a number of strategies have been proposed and are being implemented across the Pacific region. Although some adaptation measures are country-specific (for example, developing legislation around cultural practices such as kava-drinking to protect against water-borne diseases, or experimenting with drought- and salt-resistant taro and cassava crops), the majority may be grouped under broad categories aligned with the abovementioned vulnerabilities. These measures include

Ensuring that health and safety considerations are incorporated into adaptation activities across sectors (“Health in All Policies” approach)
Improving the safety and security of food and water
Improving sanitation and hygiene facilities
Increased resourcing for health emergency risk management
Climate-proofing key health and safety infrastructure
Enhanced surveillance targeting climate-sensitive diseases and their risk sources
Applied environmental epidemiological research focusing on climate-sensitive diseases
New and improved communication pathways between the health sector, meteorology services and other stakeholders, including trialing and evaluating climate-based early warning systems.

## Discussion

The climate change and health vulnerability assessment and adaptation planning project in the Pacific is similar in some respects to the corresponding work being carried out in other regions (Brooks and Adger 2003; Confalonieri et al. 2009; Wolf et al. 2014). However, there are some significant differences in terms of the process, findings, and implications that distinguish climate change and health issues in PICs from those in other countries of the world.

In terms of process, the precise methods by which the assessments were performed and adaptations planned varied from country to country. These included highly focused, largely quantitative assessments in the Marshall Islands and FSM, as distinct from a more deliberative, qualitative process employed in Nauru, Solomon Islands, and Vanuatu, where a modified environmental health impact assessment approach was employed (Spickett and Katscherian 2014; Spickett et al. 2013). In Kiribati, a mixed-methods, “middle way” approach proved effective in combining a review and analysis of the available data with a pragmatic process of inter-agency collaboration and stakeholder engagement, which has contributed to Kiribati’s NCCHAP being among the first to undergo government ratification and implementation (McIver et al. 2014).

With respect to outcomes, the issue of NCDs, in particular, was of unprecedented prominence in the Pacific in the face of climate change. With PICs already experiencing the highest rates of NCDs in the world (Mannava et al. 2015), the potential for climate change to act as an additional driver of NCD risk is considerable and of significant concern.

**Figure 3:**
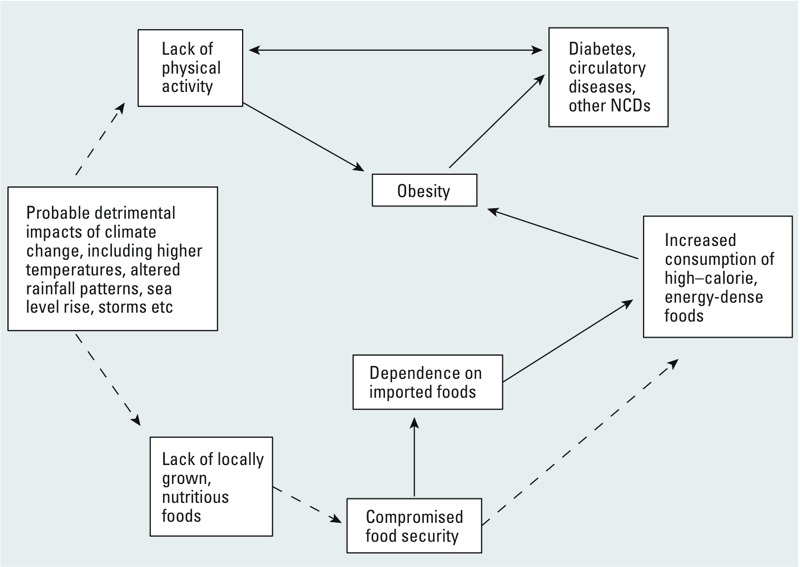
Conceptual model summarizing the pathways between climate change and NCDs (broken arrows represent hypothetical links).

Although the literature on climate change and NCDs is relatively scant and has hitherto focused primarily on the implications of heat for individuals with preexisting NCDs (Friel et al. 2011; Kjellstrom and McMichael 2013; Kovats and Hajat 2008; Shubair et al. 2013), in the Pacific region, there is a very real concern that climate change may act as an additional risk factor for NCDs. It is likely that the Pacific region is—or will be—the first to experience the consequences of the interaction between climate change phenomena and other factors driving the burden of NCDs, such as physical inactivity, food insecurity, and poor nutrition. The schema in Figure 3, developed in consultation with the climate change and health team in Nauru (a tiny Pacific island country with one of the highest burdens of NCDs in the world), summarizes these interactions as they are perceived in a number of countries across the region.

It must also be acknowledged that PICs are very likely to be among the first communities to be forced to relocate because of anthropogenic climate change (Campbell 2014). There is some evidence that this forced migration—both internal and external—is already taking place (Birk and Rasmussen 2014; Locke 2009), and the physical and psychosocial health consequences of this phenomenon must not be underestimated (Butler et al. 2014; Reuveny 2007).

PICs face substantial challenges in implementing plans for adaptation. They include the scarce resources available to health sectors that are typically already under significant strain. Shortfalls in data, information systems, human resources, technical capacity, infrastructure, and finance are the rule, rather than the exception, in the Pacific region.

In light of the stark realities described above, many of the adaptation strategies recommended over the course of this climate change and health project were explicitly considered in terms of their overall utility, applicability, and feasibility in the context of profoundly underresourced health systems. Thus, the theoretical requirement for “additionality” mentioned in the international climate change and health literature with respect to adaptation (Füssel 2007) was considered significantly less important for health systems support in the Pacific than pragmatic, realistic measures that would both improve health care and build health systems resilience to climate change. Examples of these measures include improving water, sanitation, and hygiene systems and scaling up vector control. Such interventions have clear, broad, and long-term benefits, climate change notwithstanding, but they may not be possible for small, developing countries to implement without the avenues for resources and technical support afforded by adaptation.

There may, however, be some modest advantages for PICs in adapting to climate change. Principal among these advantages is the clear consensus about the need for action: debate about the science and implications of climate change is redundant in these countries, which are already experiencing its impacts. In addition, the small size of most PICs, where populations range from approximately 10,000 in Nauru and Tuvalu to < 1,000,000 in Fiji (Table 1), and the close-knit nature of such small, isolated communities, enables a relatively high degree of collaboration on adaptation between sectors, which has the potential for increased agility in decision making. There is some indication, however faint, that it may yet prove somewhat easier to achieve coherence in climate change and health governance in relatively cohesive Pacific island communities with shared traditional values than in other countries at varying levels of development but with looser or weaker social capital (Adger 2001; Bowen et al. 2013; Woodward et al. 2000).

There are clear limits to the effectiveness of adaptation, some of which will be tested even if, as is hoped, effective climate change mitigation policy is soon agreed upon and implemented at the global scale. Perhaps the clearest example is that of sea level rise, which threatens the very existence of low-lying island communities, posing an existential threat to the atoll nations of Kiribati, Marshall Islands, and Tuvalu (see Table 1).

One of the most promising areas of potential benefit, from both an economic and a social perspective, lies in co-benefits—the health gains anticipated from action on climate change mitigation—which are most pertinent in relation to NCDs (Ganten et al. 2010). Well-chosen disease-prevention strategies, such as decreased fossil fuel use, increased active transport (e.g., walking and cycling), and greater consumption of fresh, local foods instead of imported products, have obvious health benefits and will help reduce the pressure on the world’s climate.

It is impossible to address vulnerability and adaptation to climate change in the Pacific without pointing out the gross inequities and injustice that are involved. Pacific island countries have made infinitesimal contributions to the planetary problem of anthropogenic climate change, yet they will be among those who suffer most from its consequences. Industrialized countries have a clear responsibility to both scale up mitigation efforts to arrest climate change and to provide the necessary financial, technical, and in-kind support to developing countries to strengthen their coping capacity via adaptation in the meantime.

Finally, recognizing that PICs are among many countries in the world battling climate change as one of a number of significant impediments to social, economic, and health development, WHO is in the process of providing detailed guidance, in the form of frameworks, to assist member states in scaling up essential public health packages for health adaptation and building climate-resilient health systems (Neira 2014).

## Conclusion

Pacific island countries are among the most vulnerable societies in the world to the health impacts of a changing climate.

Managing these health risks will require frequent revisions of adapation plans to take into account post-implementation reviews; new knowledge and understanding of climate change and health processes, pathways, and risks; and changes in relevant aspects of Pacific societies such as institutional structures, economic development, technology, and demographics.

This paper, and the corresponding WHO report released in early 2016, represent the first comprehensive synthesis of the current state of knowledge of health and climate change in the Pacific islands. This is but the first, important step in a long journey, for which PICs will require substantial and ongoing support.
